# Transudative Pleural Effusion as a Rare Manifestation of Disseminated Tuberculosis With Tubercular Myocarditis: A Case Report

**DOI:** 10.7759/cureus.108863

**Published:** 2026-05-14

**Authors:** Rudra P Samanta, Srikant Agarwal, Saurabh Pathak, Sangita Kamath

**Affiliations:** 1 Pulmonology, Tata Main Hospital, Jamshedpur, IND; 2 Nephrology, Tata Main Hospital, Jamshedpur, IND; 3 General Medicine, Tata Main Hospital, Jamshedpur, IND

**Keywords:** chronic granulomatous inflammation, disseminated tuberculosis, light's criteria, mycobacterium tuberculosis complex, thoracoscopy, transudative pleural effusion, tubercular myocarditis

## Abstract

Tuberculosis (TB) remains a major public health burden, particularly in India, and is known for its wide spectrum of clinical presentations ranging from pulmonary to extrapulmonary disease. Pleural effusion is a well-recognised complication of TB and is characteristically exudative in nature. We report a rare case of a 27-year-old immunocompetent female patient who presented with a right-sided pleural effusion that was transudative by Light's criteria. Rather than excluding TB, clinical suspicion was maintained, leading to a thorough diagnostic workup. The patient was ultimately diagnosed with disseminated TB involving the pleura, mediastinal lymph nodes, abdomen, pericardium, and myocardium. The transudative character of the effusion was attributed to cardiac failure secondary to tubercular myocarditis, evidenced by a severely reduced left ventricular ejection fraction of 30%. Thoracoscopy with pleural biopsy and mycobacterial culture confirmed the diagnosis. The patient responded well to antitubercular therapy (ATT) with complete radiological resolution of the pleural effusion. This case underscores that a transudative pleural effusion does not exclude TB and that thoracoscopy should be considered when clinical suspicion remains high despite atypical biochemical findings.

## Introduction

Tuberculosis (TB) caused by *Mycobacterium tuberculosis* complex is one of the most prevalent infectious diseases globally and remains a leading cause of morbidity and mortality in India [1]. While pulmonary TB is the most common form, extrapulmonary manifestations are well documented and can affect virtually any organ system. Pleural involvement is among the most frequent extrapulmonary presentations, accounting for up to 20-30% of cases in high-burden settings. Tuberculous pleural effusions are characteristically exudative, lymphocyte-predominant, and associated with an elevated adenosine deaminase (ADA) level, reflecting the delayed-type hypersensitivity and direct pleural space infection that underlie their pathogenesis [2].

Cardiac involvement in TB, though uncommon, carries significant morbidity and mortality. Tubercular pericarditis is the most frequent cardiac manifestation, while myocardial involvement is far rarer, estimated to account for fewer than 0.1% of TB-related deaths, and can result in dilated cardiomyopathy and congestive cardiac failure with a reported mortality of up to 30%. Disseminated TB, defined as simultaneous involvement of two or more non-contiguous organ systems, represents a particularly severe form of the disease in which such cardiac complications may co-exist with pleural, abdominal, and mediastinal involvement [3,4].

A transudative pleural effusion in the setting of TB is exceptionally rare and, when present, is generally attributable to a concurrent systemic cause of low oncotic pressure or elevated hydrostatic pressure rather than to direct pleural involvement. We describe a case in which a transudative pleural effusion was the presenting feature of disseminated TB, with the cardiac failure from tubercular myocarditis providing the haemodynamic explanation for the atypical biochemical profile. This case highlights the importance of maintaining clinical suspicion for TB even when initial investigations appear inconsistent with the diagnosis.

## Case presentation

A 27-year-old immunocompetent female patient presented to the outpatient department with a two-week history of productive cough, progressive anorexia, and exertional dyspnoea for five days, accompanied by a one-week history of low-grade fever. Her past medical history was notable for juvenile myoclonic epilepsy, for which she had been off antiepileptic medication for two years. There was no documented history of prior TB, immunosuppressive therapy, or contact with a known TB case.

On examination, she appeared pale and mildly tachypnoeic. Cardiovascular auscultation revealed normal first and second heart sounds with an audible S3 gallop, raising concern for cardiac dysfunction. The patient reported orthopnoea but did not describe paroxysmal nocturnal dyspnoea or bilateral pedal oedema. The presence of orthopnoea without the full constellation of classical heart failure symptoms further illustrates the atypical and incomplete clinical expression of the underlying cardiac dysfunction in this patient, likely reflecting the subacute and insidious onset of myocarditis-induced systolic dysfunction. Respiratory examination demonstrated dullness to percussion and diminished breath sounds at the right base, extending to the interscapular and infrascapular regions, consistent with a moderate-to-large right-sided pleural effusion. Abdominal examination revealed no organomegaly, but shifting dullness was present, indicating ascites. Neurological examination identified bilateral proximal myopathy of the lower limbs (the patient was unable to rise from a squatting position or from a chair without assistance) in the absence of sensory deficits, cerebellar signs, or abnormal deep tendon reflexes. Fundoscopy was normal.

**Figure 1:**
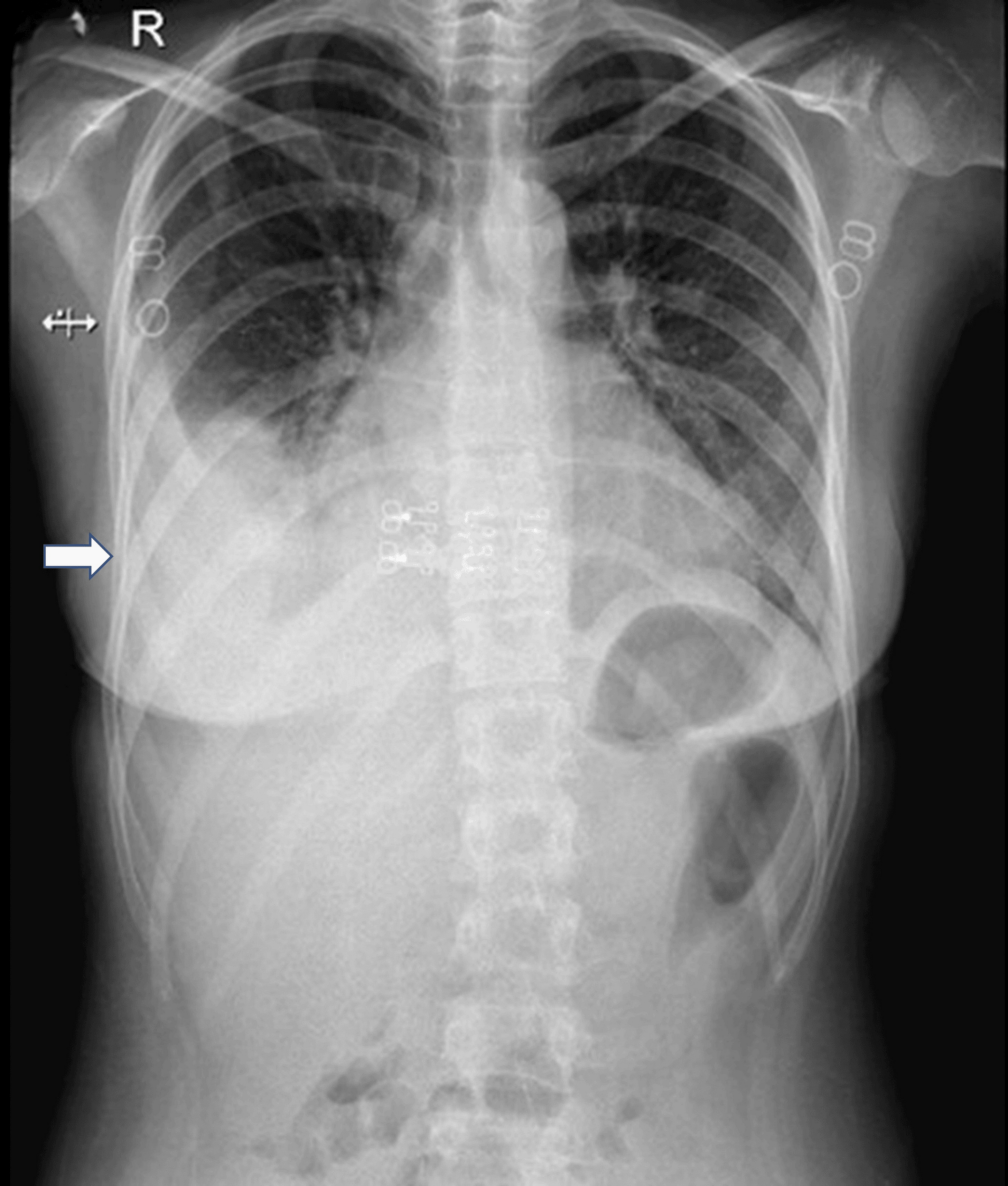
Chest radiograph (PA view) at presentation Chest X-ray (PA view) demonstrating right-sided pleural effusion. Homogeneous opacification of the right hemithorax with blunting of the costophrenic angle, consistent with a moderate-to-large pleural effusion at presentation. PA: posteroanterior

Baseline investigations revealed a haemoglobin of 5.5 g/dL (reference range: 12-16 g/dL for adult females), consistent with severe anaemia. Chest radiography confirmed a right-sided pleural effusion (Figure 1).

Diagnostic thoracocentesis was performed. Pleural fluid analysis classified the effusion as transudative by Light's criteria: pleural fluid-to-serum protein ratio of 0.45 (threshold <0.5) and pleural fluid-to-serum LDH ratio of 0.53 (threshold <0.6). Notably, the ADA level was 20.6 U/L (below the conventional TB threshold of 40 U/L), and cytology demonstrated a mixed cellularity with 70% polymorphonuclear cells and 10% lymphocytes, with a total white cell count of 5,000 cells/cumm (Table 1). Pleural fluid glucose and B-type natriuretic peptide (BNP) were not measured at the time of initial thoracocentesis. In retrospect, pleural fluid BNP, with values above 1500 pg/mL considered supportive of a cardiac aetiology, would have provided additional mechanistic corroboration for the haemodynamic basis of the transudative effusion and is recommended in future similar presentations. These findings, taken in isolation, would not typically prompt further investigation for TB; however, the clinical context necessitated a broader workup.

**Table 1:** Pleural fluid biochemistry, cardiac biomarker analysis, and follow-up cardiac function Baseline laboratory and pleural fluid findings at presentation with corresponding reference ranges, supplemented by follow-up cardiac echocardiographic data. The findings demonstrate transudative pleural effusion by Light's criteria, a low ADA level, neutrophil-predominant cytology, and associated cardiac dysfunction at presentation. Pleural fluid glucose and BNP were not measured at the time of initial thoracocentesis and are noted as recommended investigations for future similar cases. Follow-up transthoracic echocardiography demonstrated partial but significant recovery of left ventricular systolic function, with EF improving from 30% at presentation to 50% after the initiation of antitubercular therapy and cardiac failure management, consistent with reversal of myocarditis-induced cardiomyopathy. ADA: adenosine deaminase; LDH: lactate dehydrogenase; WBC: white blood cell; EF: ejection fraction; BNP: B-type natriuretic peptide; TB: tuberculosis Light's criteria: pleural effusion is classified as exudative if any of the following are present: pleural fluid-to-serum protein ratio >0.5, pleural fluid-to-serum LDH ratio >0.6, or pleural fluid LDH greater than two-thirds the upper limit of normal serum LDH; absence of all these criteria indicates a transudative effusion.

Parameter	Patient value	Reference range	Interpretation
Pleural fluid biochemistry			
Pleural fluid protein/serum protein ratio	0.45	<0.5 (transudate)	Transudative
Pleural fluid LDH/serum LDH ratio	0.53	<0.6 (transudate)	Transudative
ADA (U/L)	20.6 U/L	<40 U/L (non-TB); >40 suggestive of TB	Low
Total WBC count (cells/cumm)	5,000	<1,000 (transudate typical)	Elevated
Polymorphonuclear cells (%)	70%	<25% (typical TB lymphocyte predominant)	Neutrophilic predominance
Lymphocytes (%)	10%	>50% (typical TB)	Low
Pleural fluid glucose	Not measured	60-110 mg/dL	Not available
Pleural fluid BNP	Not measured	>1500 pg/mL supportive of cardiac transudate	Not available; recommended in future cases
Cardiac biomarkers and function			
Cardiac troponin I	0.03 ng/mL	<0.02 ng/mL	Mildly elevated
EF: at presentation	30%	55-70%	Severely reduced
EF: on follow-up	50%	55-70%	Improved; partial recovery
Pulmonary artery systolic pressure	45 mmHg	15-30 mmHg	Elevated
Left atrial size	Borderline dilated	Normal	Mild abnormality

Human immunodeficiency virus (HIV) serology was negative. Electrocardiography showed sinus tachycardia. Cardiac troponin I was mildly elevated at 0.03 ng/mL (reference range: <0.02 ng/mL). Transthoracic echocardiography revealed globally impaired left ventricular systolic function with an ejection fraction (EF) of 30%, global left ventricular hypokinesia, borderline left atrial dilatation, an elevated pulmonary artery systolic pressure of 45 mmHg, and a small patent foramen ovale (PFO) with left-to-right shunting. A normal left ventricular cavity size in the context of global hypokinesia with a markedly reduced EF is consistent with myocarditis rather than ischaemic cardiomyopathy. The cardiologist attributed the cardiac dysfunction to myocarditis, thereby providing the haemodynamic explanation for the transudative character of the pleural effusion. Cardiac failure management was initiated with carvedilol, losartan, furosemide, and spironolactone. Brain magnetic resonance imaging (MRI) was unremarkable.

**Figure 2:**
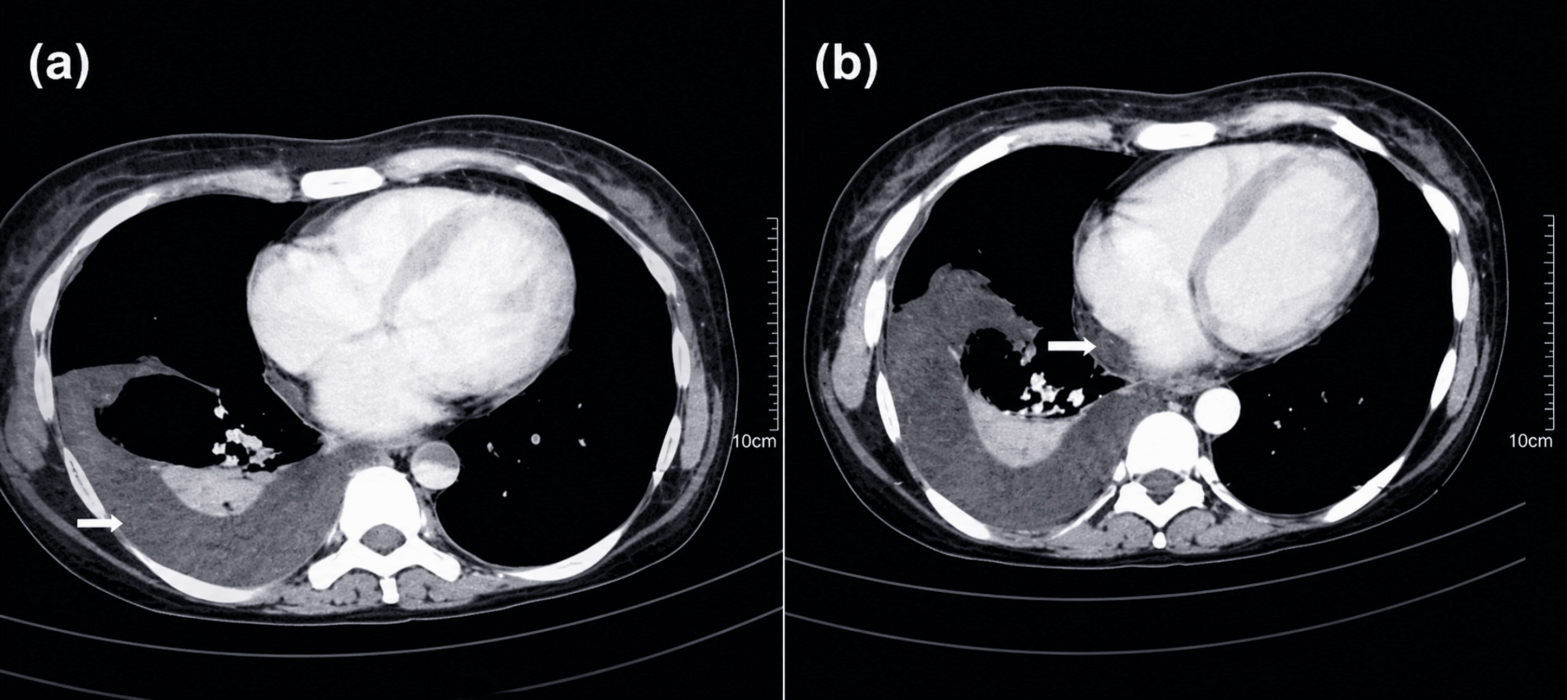
CECT of the thorax demonstrating disseminated involvement: (a) right-sided pleural effusion (arrow) and (b) small pericardial effusion (arrow) CECT images demonstrating right-sided pleural effusion and associated pericardial effusion, along with bilateral ground-glass opacities and mediastinal lymphadenopathy. Arrows indicate the areas of interest. CECT: contrast-enhanced computed tomography

Contrast-enhanced computed tomography (CECT) of the thorax demonstrated a moderate right-sided pleural effusion, bilateral ground-glass opacities, pre-tracheal and pre-vascular lymphadenopathy (largest node approximately 1 cm), and a mild pericardial effusion (Figure 2). The bilateral ground-glass opacities raised the differential of an atypical pneumonic process or, in the context of the severely reduced EF, pulmonary oedema.

CECT of the abdomen revealed circumferential wall thickening with luminal narrowing and pericolic fat stranding at the distal ileum and ileocaecal junction, consistent with ileocaecal TB, mesenteric lymphadenopathy with the largest node measuring 1 cm, a splenic infarct, and minimal free pelvic fluid. The combination of mediastinal lymphadenopathy, abdominal lymphadenopathy, ileocaecal involvement, pericardial effusion, bilateral pulmonary infiltrates, and cardiac dysfunction in a young patient from a TB-endemic region raised strong clinical suspicion for disseminated TB.

**Figure 3:**
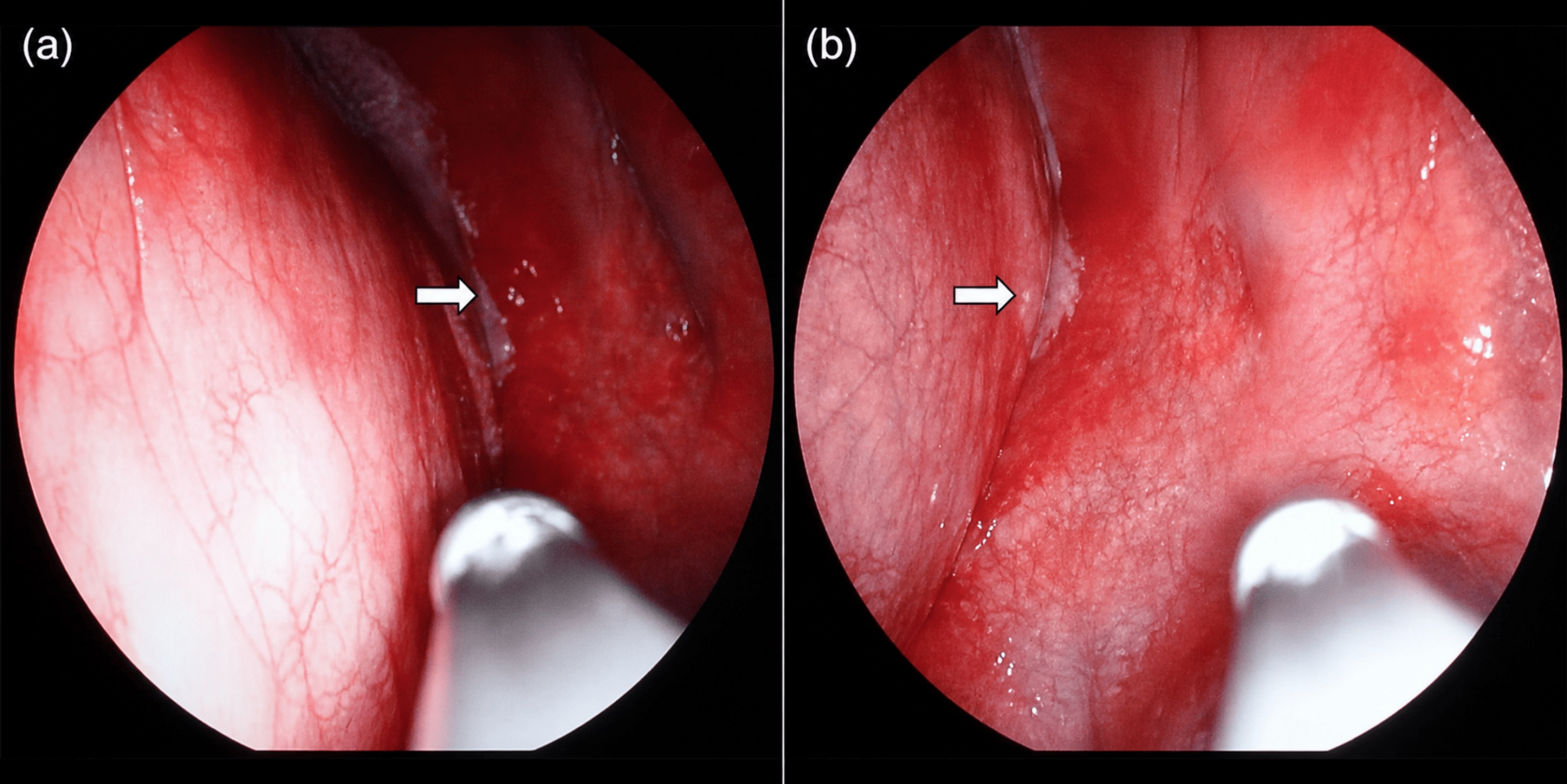
Medical thoracoscopy showing pleural involvement in disseminated tuberculosis: (a) visceral pleura showing miliary nodules (arrow) and (b) parietal pleura showing similar nodular deposits (arrow) Thoracoscopic images demonstrating multiple small whitish nodules over pleural surfaces consistent with miliary tuberculous seeding. Arrows indicate representative nodular lesions targeted for biopsy. Histopathology confirmed granulomatous inflammation and mycobacterial culture was positive for *Mycobacterium tuberculosis*.

Given the high clinical suspicion and the atypical pleural fluid biochemistry, medical thoracoscopy was performed. The visceral and parietal pleural surfaces showed scattered miliary nodules (Figure 3). Targeted pleural biopsies were obtained and sent for histopathological examination and mycobacterial culture.

Histopathology of the pleural biopsy demonstrated chronic granulomatous inflammation. GeneXpert MTB/RIF assay detected *Mycobacterium tuberculosis* (trace positive); rifampicin resistance was reported as indeterminate, likely reflecting the low bacterial load. Mycobacterial culture on Lowenstein-Jensen (LJ) medium subsequently confirmed growth of *Mycobacterium tuberculosis* complex, validating the diagnosis.

On the basis of thoracoscopic findings, CT of the abdomen features, and histopathological confirmation, a diagnosis of disseminated TB with pleural, mediastinal, abdominal, pericardial, and probable myocardial involvement was established. Antitubercular therapy (ATT) was commenced with isoniazid, rifampicin, pyrazinamide, and ethambutol in standard weight-based dosing per the Revised National Tuberculosis Control Programme (RNTCP) guidelines.

However, the patient developed drug-induced hepatotoxicity. ATT was temporarily suspended and modified to a hepatotoxicity-sparing regimen of ethambutol and streptomycin. Levofloxacin, which would ordinarily be considered as a substitute fluoroquinolone, was specifically withheld in view of her known history of juvenile myoclonic epilepsy, given the risk of lowering the seizure threshold. Once liver function tests normalised, isoniazid and rifampicin were reintroduced sequentially. Streptomycin was discontinued after two months as per protocol.

**Figure 4:**
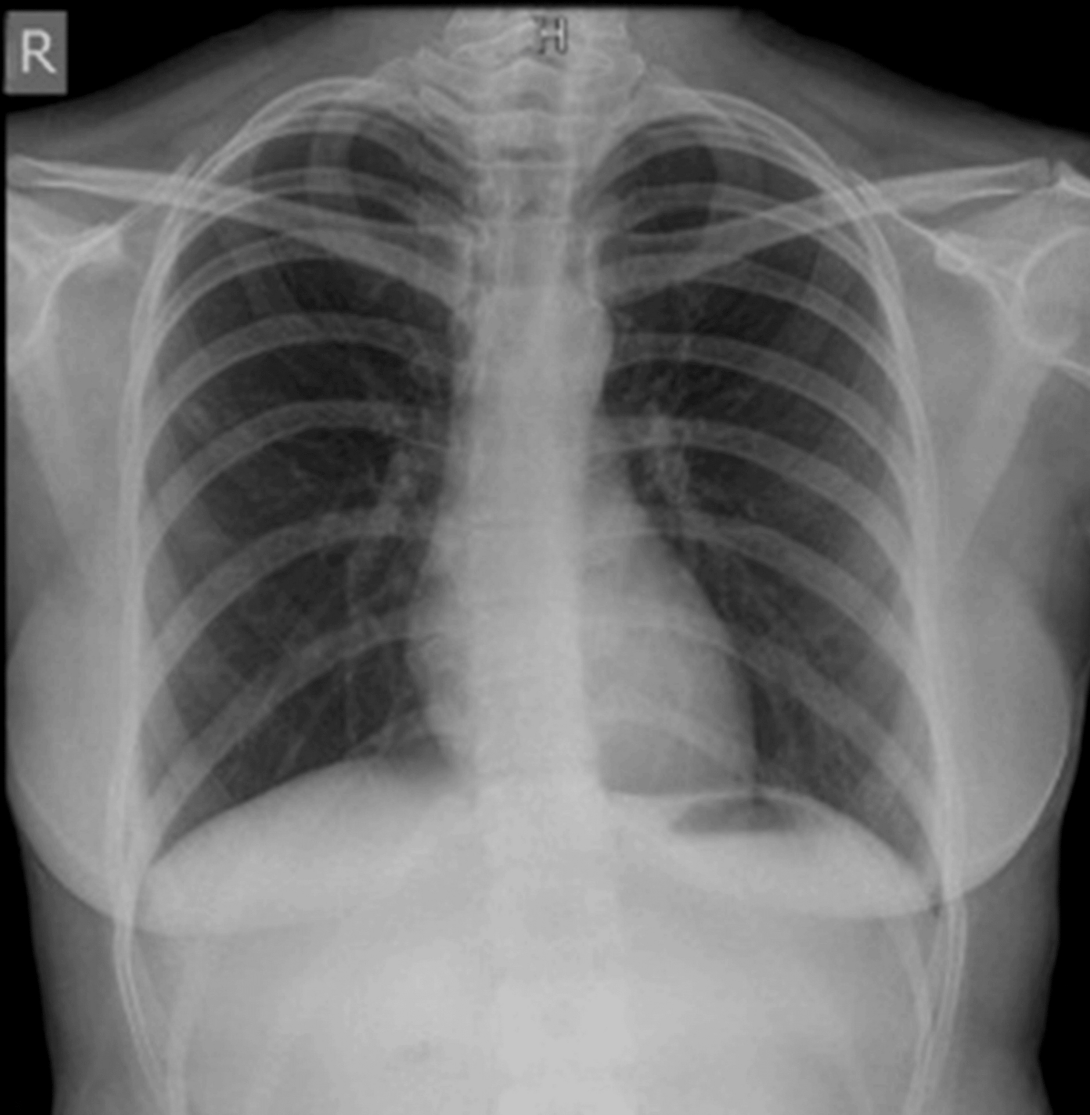
Chest X-ray (PA view) at the completion of antitubercular therapy demonstrating complete resolution of right-sided pleural effusion Follow-up radiograph confirming full radiological resolution with clearance of the pleural opacity and restoration of normal lung markings, correlating with the patient's clinical improvement. PA: posteroanterior

The patient showed progressive clinical improvement on follow-up. Repeat chest radiography at treatment completion demonstrated complete resolution of the right-sided pleural effusion (Figure 4), with symptomatic recovery and improved functional status. Repeat transthoracic echocardiography performed following the completion of ATT and ongoing cardiac failure management demonstrated significant improvement in left ventricular systolic function, with the EF recovering from 30% at presentation to 50% on follow-up. Global hypokinesia had resolved, consistent with reversal of the myocarditis-induced cardiomyopathy in response to targeted therapy. This functional cardiac recovery correlated with the patient's symptomatic improvement and restored exercise tolerance.

## Discussion

This case illustrates two compounding diagnostic rarities: a transudative pleural effusion in the setting of confirmed TB and tubercular myocarditis causing cardiac failure in a young immunocompetent patient. Each finding in isolation is unusual; their co-occurrence in the same patient is exceptional.

Pleural effusions in TB are pathophysiologically driven by a lymphocyte-mediated hypersensitivity response to mycobacterial antigens within the pleural space, as well as direct pleural infection, producing protein-rich, exudative fluid. The transudative nature of the effusion in our patient was attributable not to the pleural TB itself but to the concurrent cardiac failure from tubercular myocarditis. This mechanistic distinction is critical: the pleura was clearly involved by TB (confirmed by thoracoscopy, granulomatous histopathology, and positive mycobacterial culture), yet the haemodynamic consequence of myocarditis-induced systolic dysfunction, with an EF of 30% and elevated pulmonary venous pressures, was the dominant determinant of the effusion's biochemical character, diluting the pleural fluid proteins below the exudative threshold.

Cardiac involvement occurs in approximately 1% of TB cases, most commonly affecting the pericardium [3]. Myocardial involvement is far rarer, estimated to account for fewer than 0.1% of TB-related mortality [3]. Three histological patterns have been described: miliary tubercles of the myocardium, diffuse myocardial infiltration, and nodular tuberculomas [4,5]. Proposed mechanisms of myocardial seeding include haematogenous dissemination, lymphatic spread, and direct extension from contiguous mediastinal or pericardial structures [6]. Although endomyocardial biopsy was not performed in this patient, the echocardiographic picture of global hypokinesia with preserved left ventricular cavity dimensions, inconsistent with ischaemic cardiomyopathy, alongside evidence of disseminated TB, provides strong circumstantial evidence of tubercular myocarditis. Literature reports a 30% mortality rate for this complication, emphasising the importance of early diagnosis [7]. The recovery of left ventricular EF from 30% at presentation to 50% on follow-up echocardiography in this patient provides important evidence of the reversibility of myocarditis-induced systolic dysfunction when the underlying tuberculous aetiology is identified and treated early. This improvement occurred despite the need for temporary suspension and individualised modification of the ATT regimen due to drug-induced hepatotoxicity, highlighting that even an interrupted or modified course of ATT, when combined with guideline-directed cardiac failure management, can achieve meaningful functional cardiac recovery. Clinicians should therefore not be deterred from pursuing aggressive diagnostic evaluation and prompt ATT initiation in suspected tubercular myocarditis, even in the context of complex comorbidities or drug intolerances. Although cardiac MRI has demonstrated utility in characterising myocardial inflammation and late gadolinium enhancement in TB myocarditis [8-14], it was not performed in this case due to non-availability but should be considered wherever feasible in suspected tubercular myocarditis. On cardiac MRI, active tubercular myocarditis characteristically demonstrates high T2-weighted signal reflecting myocardial oedema and late gadolinium enhancement in a non-ischaemic pattern, typically mid-wall or epicardial, corresponding to areas of granulomatous inflammation and early fibrosis. These features are distinct from the subendocardial or transmural late gadolinium enhancement pattern seen in ischaemic cardiomyopathy, and their identification would have further supported the diagnosis and guided prognostication in this patient.

The low ADA level (20.6 U/L) in the pleural fluid is explicable in this context. ADA elevation reflects lymphocyte activation within the pleural space; in transudative effusions driven by cardiac failure, the predominant cellular response is polymorphonuclear rather than lymphocytic, and the overall protein concentration is lower, resulting in a diluted ADA value that does not reflect true pleural inflammatory activity. Clinicians should be aware that a low ADA does not exclude tuberculous pleuritis, particularly when the effusion is transudative. The absence of pleural fluid BNP measurement in this case represents a diagnostic opportunity that, in retrospect, should have been pursued. Pleural fluid BNP levels above 1500 pg/mL have been reported as a reliable marker of cardiac transudates, distinguishing them from other causes of pleural effusion including hepatic or nephrotic aetiologies. In a case such as this, where the transudative character was attributed to cardiac failure from tubercular myocarditis, a markedly elevated pleural fluid BNP would have provided early, non-invasive corroboration of the haemodynamic mechanism before echocardiography confirmed the severely reduced EF. Future cases presenting with transudative pleural effusion of uncertain aetiology should include pleural fluid BNP as part of the initial biochemical panel.

The decision to proceed to medical thoracoscopy despite transudative biochemistry was pivotal in this case. Thoracoscopy offers direct visualisation of the pleural surface and facilitates targeted biopsy, yielding a diagnostic sensitivity of over 90% for tuberculous pleuritis [2]. The characteristic miliary nodular pattern observed on the pleural surface, confirmed on histopathology as granulomatous inflammation and on culture as *Mycobacterium tuberculosis*, would not have been identified by pleural fluid analysis alone. This case argues strongly for the use of thoracoscopy in any case with sustained clinical suspicion of TB regardless of pleural fluid Light's classification.

The management of this patient required individualised modification of the standard ATT regimen. Drug-induced hepatotoxicity is a well-recognised complication of first-line ATT, particularly with isoniazid, rifampicin, and pyrazinamide. The sequential reintroduction of hepatotoxic agents after liver function recovery is standard practice [15]. The deliberate omission of levofloxacin is noteworthy: fluoroquinolones lower the seizure threshold and carry a specific caution in patients with epilepsy. Given her documented juvenile myoclonic epilepsy, the risk-benefit analysis favoured avoidance of this agent, consistent with standard prescribing caution in this population.

The concurrent proximal myopathy documented on neurological examination merits comment. While TB myopathy is rare, the finding could represent a paraneoplastic-like inflammatory process in the context of disseminated disease, direct muscular involvement, or nutritional deficiency in the setting of severe anaemia (Hb 5.5 g/dL) and prolonged illness. This was not definitively attributed to TB in the original report and warrants further investigation in similar future cases.

## Conclusions

This case demonstrates that TB can present with a transudative pleural effusion when concurrent cardiac failure secondary to tubercular myocarditis alters the haemodynamic milieu of the pleural space. The pleural fluid biochemistry, including the ADA level and Light's criteria classification, may be fundamentally misleading in this setting and should not be used as the sole criterion to exclude TB. A thorough clinical assessment, supplemented by cross-sectional imaging, echocardiography, and, crucially, medical thoracoscopy with pleural biopsy, is necessary to establish the correct diagnosis in atypical presentations.

The identification of *Mycobacterium tuberculosis *by culture remains the gold standard for confirming tuberculous pleuritis, but in high-burden settings, thoracoscopic visualisation of miliary pleural nodules with histological demonstration of caseating granulomas provides a pragmatic and reliable diagnostic pathway. GeneXpert MTB/RIF, while rapid, may yield trace-positive or indeterminate results in paucibacillary disease, and culture must be regarded as complementary rather than redundant. Furthermore, the documented recovery of left ventricular EF from 30% to 50% on follow-up echocardiography in this patient demonstrates that tubercular myocarditis-induced cardiomyopathy is potentially reversible with prompt diagnosis and treatment, even when standard ATT requires modification due to hepatotoxicity or drug contraindications.

Cardiac MRI should be incorporated into the diagnostic workup of suspected tubercular myocarditis wherever feasible. Individualised modification of ATT is essential when standard regimens are complicated by hepatotoxicity or contraindicated drugs. Further research into novel pleural biomarkers, including unstimulated pleural fluid interferon-γ, may improve diagnostic accuracy in future cases where thoracoscopy is unavailable or contraindicated.
